# The relationship between sexual activity during chemotherapy and sexual functioning after treatment among females with breast and gynecologic cancer

**DOI:** 10.1007/s00520-025-09858-z

**Published:** 2025-08-28

**Authors:** Kristen M. Carpenter, Katherine Conroy, Lora L. Black, Ritu Salani, Maryam Lustberg

**Affiliations:** 1 The Ohio State University Wexner Medical Center, Columbus, OH USA; 2 University of Washington, Seattle, WA USA; 3 University of California, Los Angeles, CA USA; 4 Yale University, New Haven, CT USA

**Keywords:** Sexual functioning, Female cancer survivor, Sexual satisfaction, Gynecologic cancer, Breast cancer

## Abstract

**Purpose:**

Sexual inactivity has been shown to be a predictor of future sexual problems and related distress in female cancer survivors; however, very little is known about the sexual lives of women in active treatment. The aims of present study are to estimate rates of sexual activity during adjuvant chemotherapy and their relationship to sexual outcomes 12 months after therapy initiation.

**Methods:**

Self-report questionnaires were completed by gynecologic and breast cancer patients (*n* = 58) prior to their first chemotherapy infusion, at three timepoints during treatment, and at follow-up 1 year later. Questionnaires assessed sexual activity, sexual desire, sexual arousal, sexual satisfaction, depressive symptoms, and treatment toxicities.

**Results:**

Engagement in sexual activity during chemotherapy was significantly associated with reports of experiencing sexual desire (*B* = 1.94, *p* = .03), arousal (*B* = 3.31, *p* = .01), and higher satisfaction ratings (*β* = .33, *p* = .02) 12 months after the initiation of chemotherapy.

**Conclusions:**

The results indicate that remaining sexually active during treatment is associated with better sexual function and higher sexual satisfaction in the year following chemotherapy initiation.

## Introduction

Treatments for breast and gynecologic cancer are associated with a variety of sexual problems [1, 2] often reported at higher rates than would be expected based on reports from the general population [3–5]. The results of recent cross-sectional and longitudinal cohort studies suggest that a majority of breast cancer survivors experience some degree of sexual dysfunction or lower sexual activity engagement [6–8]. Among female cancer survivors, the most highly endorsed sexual difficulty is low desire, with many survivors reporting multiple problems [9–13].

Given high rates of sexual complaints in cancer survivors, it is unsurprising that after completing treatment, approximately 30–60% of breast and gynecologic cancer survivors are sexually inactive [10–14]. While sexual inactivity is not specific to female cancer survivors, the reasons for inactivity vary between those with and without a history of cancer. Gynecologic cancer survivors cite physical problems as the most common reason for sexual inactivity [11], whereas post-menopausal females without a history of cancer cite not having a partner [15]. When looking specifically at female cancer survivors with partners, the majority attribute their sexual inactivity to intrapersonal (e.g., pain, vaginal dryness, lack of interest, and fatigue) versus interpersonal (e.g., partner unavailability) factors [12]. These findings point to the significant and unique impact of cancer treatment on patients’ sexual lives above and beyond common reasons for sexual inactivity, such as menopausal symptoms or partners [15–17].

Studies have shown that sexual inactivity is associated with lower sexual satisfaction among female cancer survivors regardless of current cancer or treatment status [12] and the general population [16, 17]. In addition, sexual inactivity is associated with feeling unattractive, feeling less “like a woman,” (as applicable) and experiencing depressive symptoms among female cancer survivors [12]. Given these findings, sexual inactivity may serve as a useful predictor of future sexual problems and psychosocial distress in female cancer survivors, though little is known about the sexual lives of females in active treatment. Patients undergoing chemotherapy experience a variety of toxicities (e.g., fatigue, nausea, and neuropathy) that interfere with a variety of activities of daily living, including sexual activity and function [18, 19]. Many patients experience both “good” and “bad” days; recommendations for leisure and other activities often amount to “as tolerated” for those undergoing treatment, but it is well established that continued engagement with such activities is associated with a more favorable quality of life trajectory [20–22]. However, sexual activity has not traditionally been discussed in similar ways, and many patients rarely remember if sexual risks during treatment were ever discussed with providers [23]. Gaining a better understanding of sexual activity during treatment and its relationship to later sexual functioning may help guide clinical conversations between providers and patients before and during treatment.

## Objectives

This study was conducted as a secondary data analysis from a larger study investigating coping mechanisms and the experiences of a variety of chemotherapy side effects during treatment among females with breast and gynecologic cancer. The aims of this secondary data analysis are to (1) estimate rates of sexual activity during active treatment (i.e., adjuvant chemotherapy) in a sample of female breast and gynecologic cancer patients and (2) examine differences in sexual outcomes (sexual satisfaction and sexual function) 12 months after the initiation of chemotherapy between participants that were sexually active at some point during the treatment and those that were not. We hypothesize that a greater proportion of those that engaged in sexual activity during chemotherapy would report experiencing sexual desire and arousal 12 months after initiating chemotherapy. We also hypothesize that those who were sexually active during chemotherapy would have higher sexual satisfaction rates 12 months post chemotherapy initiation than those who were not sexually active.

Previous research has shown that several factors are related to sexual functioning among female cancer survivors [24–26]. Thus, it is important to examine these factors as possible confounds in the relationship between sexual activity and outcomes in this study. Given the number of participants in the study, exploratory analyses will be conducted in addition to primary analyses to explore whether any relationship between being sexually active during treatment and sexual outcomes 12 months after chemotherapy initiation were significant when controlling for these factors to gain better insight into these possible relationships. We hypothesize that being sexually active during chemotherapy will be associated with higher rates of sexual desire and arousal, as well as better sexual satisfaction at 12 months post chemotherapy initiation when controlling for a variety of additional factors traditionally associated with sexual functioning, In addition, we explore whether those that were sexually inactive throughout the duration of the study differed from those that engaged in sexual activity on baseline measures to better understand additional factors that may play a role in sexual functioning. We hypothesize that those who were sexually inactive during the study would have higher rates of depressive symptoms and lower relationship satisfaction ratings at baseline.

## Study design

### Participants

In the larger study, participants were cisgender female patients with gynecologic or breast cancer treated at a comprehensive cancer center associated with a midsized midwestern academic medical center. The study staff (research coordinator and trained research assistant) reviewed patient medical charts to identify eligibility for this larger study. Eligible patients were females diagnosed with stage I–III breast or gynecologic cancer, scheduled to receive chemotherapy, and could read/write English. Exclusion criteria included: prior cancer diagnosis (i.e., recurrence); Age < 21 and > 80; serious mental illness (e.g., psychosis, active addiction, and current major depressive episode); and cognitive deficit that would impact their ability to complete the interview. Eighty participants consented to participate in the larger study, but one participant was unable to complete baseline questionnaires and was thus not included in primary or secondary analyses (total *n* for larger study = 79).

We used a prospective, longitudinal design with repeated measures in the larger study. Participants were accrued following surgery (as applicable) and prior to beginning chemotherapy (e.g., within weeks of initial diagnosis). The study’s research coordinator and trained research assistant consented and approached patients and completed informed consent for the larger study in person at a clinic appointment (IRB protocol 2015C0085). As part of the consent process, participants were informed that they could skip any questions or withdraw from the study at any time without an impact on compensation, eligibility to complete the remainder of the study, or the care they received at the study institution. Participants completed assessments at baseline (prior to initiation of chemotherapy) on day one of each chemotherapy cycle (up to six cycles) and 12 months after beginning treatment with the coordinator or research assistant as part of the larger study. For the baseline sexuality assessments, participants were asked during assessment administration by the research assistant to answer based on their experiences prior to when chemotherapy symptoms may have impacted their sexual functioning. Whenever possible, participants completed assessments in full during a clinic visit. When time or distance did not allow, participants completed the interview over the phone or via mail with the coordinator or research assistant. As part of the larger study, patients were paid $35 for baseline and 12-month assessments, and $15 for each brief assessment during chemotherapy infusions. Additional information about the larger study procedures is described in previous publications [27, 28].

Of the 79 participants who completed the baseline assessment in the larger study, 58 (73%) completed the 12-month follow-up. Common causes of missing 12-month data were lost to follow-up (*n* = 14), discontinuation of chemotherapy (*n* = 4), and disease recurrence (*n* = 3). We analyzed data from the 58 participants who completed both baseline and 12-month post chemotherapy sexual health questionnaires for this secondary data analysis. Specifically, the secondary data analysis included patient responses and medical chart review from the baseline, 4-month, 8-month, and 12-month questionnaires. Data obtained via interview/survey responses, Derogatis self-report inventories, relevant medical chart data (e.g., diagnosis and treatments), and medical evaluations from the larger study were analyzed for this secondary data analysis.

### Primary analysis variables

#### Sexual activity

The presence/absence of sexual activity was assessed through two self-report questions. The first question assessed engagement in any sexual activity (either partnered or unpartnered) via a yes/no question (e.g., “Did you engage in any sexual activity this month [or since last assessment?”]). The second question assessed engagement in intercourse via a Likert scale question asking participants to indicate how often they have engaged in “intercourse (or equivalent intimate activity)” in the last month (or since last assessment).” Response options ranged from “activity has not occurred” to “four or more times per day.” Participants were categorized into either sexually “active” or “inactive” categories based on their responses to the sexual activity items administered at baseline and at the chemotherapy infusion visits. If at any of these assessments, the participant reported some sexual activity (by either answering “yes” to the first question or selecting any amount of engagement in intercourse for the second question), they were categorized as “sexually active.”

#### Sexual function

The presence/absence of current sexual desire, arousal, and orgasm was assessed via single items (i.e., *Did you experience sexual [desire, arousal, orgasm] over the past month or since last assessment*?). While orgasm was initially explored as a functional outcome alongside desire and arousal, no sexually inactive participants reported experiencing orgasm; therefore, this variable could not be included in analyses.

#### Sexual satisfaction

The global satisfaction item was taken from the Derogatis Sexual Function Inventory. Participants rated their sexual lives as a whole on a 9-point Likert scale from 0 = *could not be worse* to 8 = *could not be better* [29] over the past month or since last assessment.

### Exploratory analyses variables

Variables possibly associated with sexual outcomes were identified based on prior research [24–26] and included depressive symptomology, treatment toxicities present 12 months after chemotherapy initiation (e.g., pain and fatigue), and sociodemographic variables (e.g., household income, education, and employment status) (see Table 1). Of note, all participants identified as heterosexual, and thus sexual orientation was not included in any model.

**Table 1:** Descriptive characteristics of the sample by sexual activity during chemotherapy (*n* = 58)

Variable	*Total M(SD)*/*n*(%)	*Sexually active M(SD)*/*n*(%)	*Sexually inactive M(SD)*/*n*(%)	*Bivariate statistic*
Age	59 (9)	57.12 (1.97)	61.22 (8.14)	*t*(56) = − 1.72, *p* =.09
Race*				
White	55 (95%)	24 (92%)	31 (96.9%)	*χ*^2^(1) =.61, *p* =.044
African American/Black	3 (5%)	2 (8%)	1 (3.1%)	*χ*^2^(1) =.61, *p* =.044
Native American	2 (3%)	2 (6%)	0 (0%)	*χ*^2^(1) = 1.68, *p* =.20
Employment status				*χ*^2^(3) = 3.32, *p* =.35
Employed (full-time or part-time)	24 (41%)	8 (31%)	16 (50%)	
Disabled	28 (48%)	16 (62%)	12 (38%)	
Temporarily unemployed	3 (5%)	1 (4%)	2 (6%)	
Retired	3 (5%)	1 (4%)	2 (6%)	
Education				*χ*^2^(1) =.28, *p* =.60
Obtained college degree	29 (50%)	14 (54%)	15 (47%)	
Did not obtain college degree	29 (50%)	12 (29%)	17 (53%)	
Marital/partnered status				*χ*^2^(4) = 2.71, *p* =.61
Single, never married	4 (7%)	2 (8%)	2 (6%)	
Currently married	37 (64%)	19 (73%)	18 (56%)	
Not married, but in a relationship	4 (7%)	1 (4%)	3 (9%)	
Separated or divorced	11 (19%)	3 (12%)	8 (25%)	
No longer married, widowed	2 (3%)	1 (4%)	1 (3%)	
Annual household income				*χ*^2^(4) = 7.12, *p* =.13
< $25,000	6 (10%)	1 (4%)	5 (16%)	
$25,000–$50,000	12 (21%)	4 (15%)	8 (25%)	
$50,001–$100,000	15 (26%)	5 (19%)	10 (31%)	
$100,001 +	20 (35%)	13 (50%)	7 (22%)	
Don’t know/prefer not to answer	5 (9%)	3 (12%)	2 (6%)	
Cancer type				*χ*^2^(2) = 9.19, *p* =.01
Breast	30 (52%)	19 (73%)	11 (34%)	
Endometrial/Uterine	16 (28%)	3 (12%)	13 (41%)	
Ovarian	12 (21%)	4 (15%)	8 (25%)	
Received surgery	56 (97%)	24 (92%)	32 (100%)	*χ*^2^(1) = 2.55, *p* =.11
Received hormonal therapy	18 (31%)	8 (31%)	10 (31%)	*χ*^2^(1) = 1.26, *p* =.53
Received radiation therapy	28 (48%)	12 (46%)	16 (50%)	*χ*^2^(1) =.09, *p* =.77

^*^ Participants were able to choose more than one racial identity

#### Depressive symptoms

The Iowa short-form of the Center for Epidemiological Studies Depression Scale (CES-D) [30] was used to assess current symptoms of depression. This short form consists of 11 items rated on 3-point Likert scales from 0 = *hardly ever or never* to 2 = *much or most of the time.* Scores can range from 0 to 22 and a cutoff score of 16 is used to assess for potential depressive symptoms.

#### Treatment toxicities

A subset of the NCI Common Terminology Criteria for Adverse Events (CTCAE), v4.0, was used [31]. Data were abstracted from electronic medical records (nurse ratings, lab and exam results, and narrative notes). Items are grouped within 26 body categories. Scores are rated on a 6-point Likert scale from 0 = *no symptoms* to 5 = *death*. We obtained subscale scores for each bodily system by averaging the items; these subscale scores were summed to obtain an overall toxicity score that ranged from 0 to 130.

#### Relationship satisfaction

Happiness in the participants’ current relationship was assessed via a single item from the Dyadic Adjustment Scale [32]. Participants were asked to indicate the phrase that best described their degree of happiness in their relationship over the past month or since the last assessment on a seven-point Likert scale ranging from 0 = *extremely unhappy* to 6 = *perfect.* Participants not in relationships were able to select “not applicable.”

### Primary analytic strategy

Chi-square and *t*-tests were used to investigate differences in sexual outcomes between those that were sexually active during the study and those that were not. Specifically, chi-square analyses were conducted to examine whether there was a difference in proportions between the two groups on reports of experiencing desire and arousal at 12 months post chemotherapy initiation. Between group *t*-tests were conducted to examine the differences in sexual satisfaction ratings 12 months after initiating chemotherapy between the two groups. Complete case analysis with no imputation was used for all analyses.

### Exploratory analytic strategy

To identify potential covariates for the exploratory aims, preliminary analyses for the exploratory aims included chi-square and independent samples *t*-tests examining between-group differences in sexual outcomes 12 months after the initiation of chemotherapy (breast vs. gynecologic cancer survivors; sexually active vs. inactive). Additional preliminary analyses were conducted to assess possible relationships between the other treatments (e.g., surgery, hormonal therapy, and surgery) and outcomes. As none of these preliminary analyses were significant, we did not include these as covariates in the exploratory model.

Regression analyses were used to assess the relationships between sexual activity during the study and sexual outcomes 12 months post chemotherapy initiation. The dichotomous functional outcomes (desire and arousal) were examined using logistic regression, and satisfaction was examined using multiple hierarchical linear regressions. In all regression models, the first step included baseline sociodemographic variables (age, partner status, and diagnosis) and baseline scores on the outcome of interest (e.g., baseline desire included in the model predicting 12-month post chemotherapy initiation desire). Step 2 included concurrent (12-month post chemotherapy initiation) depressive symptoms and treatment toxicity. In step 3, we tested whether sexual activity during treatment was associated with each outcome above and beyond the contribution of covariates. Exploratory analyses were conducted examining the subset of participants who reported no sexual activity across the entire 12-month study period. We examined correlations between demographic and health covariates and membership in this “inactive-all-year” group.

## Findings

### Sample description

Participants were majority white (95%) and currently married (64%), with an average age of 59 years. Fifty-two percent of participants were being treated for breast cancer, 28% for endometrial or uterine cancer, and 21% for ovarian cancer. Baseline sociodemographic and descriptive statistics of all relevant physical, psychosocial, and sexual variables at baseline, 4 months, 8 months, and 12 months following chemotherapy initiation can be found in Tables 1 and 2.

**Table 2:** Physical, psychosocial, and sexual variables of sample (*n* = 58)

	Baseline	4-month	8-month	12-month
	*M(SD*/*n*(%)	*M(SD)*/*n*(%)	*M(SD)*/*n*(%)	*M(SD)/n*(%)
**Physical**				
Treatment toxicity (NCI CTCAE v4) Sum Score	.95 (.63) Range 0–2.4 Breast.49(.48) Gyn.98(.62)	1.87 (.73) Range.27–3.33 Breast 1.54 (.73) Gyn 1.68 (.75)	1.44 (.81) Range.21–3.15 Breast 1.18 (.89) Gyn1.15 (.71)	1.67 (1.21) Range.27–6.17 Breast 1.60 (1.37) Gyn 1.21 (1.0)
**Emotional/psychological**				
Depression (CES-D)	5.56 (3.45) Range 0–14 Breast 5.07 (3.12) Gyn 6.07 (3.76)	5.30 (3.87) Range 0–17 Breast 4.55 (3.46) Gyn 6.07 (4.18)	3.82 (4.49) Range 0–12 Breast 3.93 (3.70) Gyn 3.68 (3.27)	4.28 (3.89) Range 0–19 Breast 3.87 (3.54) Gyn 4.71 (4.26)
**Sexual outcomes**				
Sexually active (% yes)	18 (32.7%) Breast 17 (56.7%) Gyn 1 (3.6%)	15 (25.9%) Breast 10 (33.3%) Gyn 5 (17.9%)	21 (36.2%) Breast 13 (43.3%) Gyn 8 (28.6%)	27 (46.6%) Breast 17 (56.7%) Gyn 10 (35.7%)
Experienced desire (% yes)	28 (50.9%) Breast 23 (76.7%) Gyn 5 (17.9%)	23 (39.7%) Breast 16 (53.3%) Gyn 7 (25%)	27 (46.6%) Breast 17 (56.7%) Gyn 10 (35.7%)	33 (56.9%) Breast 21 (70%) Gyn 12 (42.9%)
Experienced arousal (% yes)	22 (40.0%) Breast 19 (63.3%) Gyn 3 (10.7%)	21 (36.2%) Breast 14 (46.7%) Gyn 7 (25%)	29 (50.0%) Breast 18 (60.0%) Gyn 11 (39.3%)	33 (56.9%) Breast 21 (70.0%) Gyn 12 (42.9%)
Sexual satisfaction	3.43 (2.22) Range 0–8 Breast 3.87 (2.16) Gyn 2.92 (2.23)	3.34 (2.48) Range 0–8 Breast 3.34 (2.19) Gyn 3.33 (2.12)	3.61 (2.16) Range 0–8 Breast 3.67 (2.37) Gyn 3.55 (1.92)	3.70 (2.40) Range 0–8 Breast 4.04 (2.44) Gyn 3.35 (2.35)

### Preliminary results

Regarding group differences between diagnoses, a higher proportion of breast cancer survivors reported experiencing desire (*χ*^2^(1, *N* = 58) = 4.35, *p* = 0.037, *OR* 3.11, 95% *CI* [1.06, 9.18]) and arousal (*χ*^2^(1, *N* = 58) = 4.35, *p* = 0.037, *OR* 3.11, 95% *CI* [1.06, 9.18]), compared to gynecologic cancer survivors 12 months after chemotherapy initiation.

### Primary results

Twenty-six of the 58 participants (44.8%) reported being sexually active at one point during the year-long study. A greater proportion of sexually active participants reported experiences of desire (*χ*^2^(1, *N* = 58) = 19.15, *p* < 0.001, *OR* 16.87, 95% *CI* [4.09, 69.53]) at 12 months after initiating chemotherapy, compared to the inactive group. The crosstabulation showed that 88.5% of sexually active participants reported experiencing desire vs. 31.3% of sexually inactive participants 12 months post chemotherapy initiation. Similarly, a greater proportion of sexually active participants reported experiences of sexual arousal (*χ*^2^(1, *N* = 58) = 24.10, *p* < 0.001, *OR* 30.67, 95% *CI* [5.98, 157.37]) at 12 months after initiating chemotherapy, compared to the inactive group. The crosstabulation showed that 92.3% of sexually active participants reported experiencing desire vs. 28.1% of sexually inactive participants 12 months post chemotherapy initiation. Sexually active participants also reported significantly higher sexual satisfaction (*M* = 4.80, *SD* = 1.92), compared to sexually inactive participants (*M* = 2.76, *SD* = 2.40, *t*(52) = 3.42, *p* = 0.001, Cohen’s *d* = 0.93).

### Exploratory results

Engagement in sexual activity during chemotherapy was significantly associated with presence of sexual desire (*B* = 1.94, *p* = 0.03) and arousal (*B* = 3.31, *p* = 0.01) at 12 months following initiation of chemotherapy when controlling for covariates (i.e., baseline demographics, baseline scores of the outcome variable, 12-month depressive symptoms, and 12-month treatment toxicity ratings). Sexual activity (*β* = 0.33, *p* = 0.02) was also associated with higher sexual satisfaction scores at 12 months after chemotherapy initiation when controlling for the covariates. See Table 3 for results.

**Table 3:** Exploratory regression analysis results

	Desire*				Arousal*				Satisfaction**		
	*B(SE B)*	*Wald*	*p-value*	*OR*	*B(SE B)*	*Wald*	*p-value*	*OR*	*β*	*p-value*	*ΔR*^*2*^
Step 1	*Nagelkerke R*^*2*^ = *0.34*				*Nagelkerke R*^*2*^ = *0.28*				*F(4,43)* = *5.29, p* = *0.001 R*^*2*^ = *0.33*		
Age	−0.05(0.04)	1.11	0.30	0.96	−0.06(0.05)	1.80	0.18	0.94	-.03	0.79	
Partner Status (Y/N)	−0.79(0.78)	0.01	0.92	0.93	0.36(0.75)	0.23	0.63	1.43	0.09	0.48	
Breast Cancer (Y/N)	−0.79(0.90)	0.77	0.38	0.46	−0.37(0.77)	0.23	0.64	0.69	0.03	0.84	
Baseline Measurement	2.68(0.94)	8.13	< 0.01	14.54	2.07(0.86)	5.83	0.02	7.93	0.55	< 0.01	
											*0.33*
Step 2	*Nagelkerke R*^*2*^ = *0.35*				*Nagelkerke R*^*2*^ = *0.31*				*F(6,41)* = *3.90, p* = *0.004 R*^*2*^ = *0.36*		
Age	−0.05(0.05)	1.35	0.25	0.95	−0.07(0.05)	2.27	0.13	0.93	−0.04	0.75	
Partner Status (Y/N)	−0.21(0.81)	0.07	0.80	0.81	0.24(0.77)	0.10	0.75	1.27	0.07	0.60	
Breast Cancer (Y/N)	−0.73(0.90)	0.66	0.42	0.48	−0.27(0.78)	0.12	0.73	0.76	0.02	0.91	
Baseline Measurement	2.57(0.98)	6.93	< 0.01	13.05	1.81(0.88)	4.17	0.04	6.08	0.51	< 0.01	
CES-D	−0.08(0.10)	0.66	0.42	0.92	−0.11(0.10)	1.34	0.25	0.90	−0.19	0.15	
NCI CTAE	0.01(0.34)	< 0.01	0.97	1.01	0.13(0.35)	0.13	0.72	1.13	0.04	0.76	
											0.03
Step 3	*Nagelkerke R*^*2*^ = *0.45*				*Nagelkerke R*^*2*^ = *0.51*				*F7,(7,40)* = *4.63, p* < *0.001 R*^*2*^ = *0.45*		
Age	−0.03(0.05)	0.50	0.48	0.97	−0.08(0.05)	1.94	0.16	0.93	0.03	0.81	
Partner Status (Y/N)	−0.50(0.87)	0.33	0.57	0.61	−0.37(0.90)	0.17	0.68	0.69	0.05	0.67	
Breast Cancer (Y/N)	−1.05(1.03)	1.04	0.31	0.35	−0.18(0.93)	0.04	0.85	0.84	−0.04	0.73	
Baseline Measurement	2.15(1.10)	3.87	.05	8.57	−0.27(1.29)	0.04	0.83	0.76	0.42	< 0.01	
CES-D	−0.07(0.10)	0.52	0.47	0.93	−0.17(0.12)	2.07	0.15	0.84	−0.17	0.19	
NCI CTAE	−0.05(0.38)	0.02	0.89	0.95	0.05(0.39)	0.02	0.90	1.06	0.01	0.93	
Sexually Active (Y/N)	1.94(0.87)	4.91	0.03	6.94	3.31(1.24)	7.17	0.01	27.35	0.33	0.02	
											0.08

^*^Dichotomous outcome variable/logistic regression and

^**^continuous outcome variable/linear regression

Of the women who were sexually inactive during chemotherapy, a majority (81%) did not resume sexual activity by the 12-month follow-up. Being sexually inactive for the entire year following chemotherapy treatment initiation was associated with higher baseline levels of depressive symptoms (*r* = 0.30, *p* < 0.01) and, in partnered participants, lower baseline relationship satisfaction (*r* =  − 0.51, *p* < 0.01).

## Discussion

Nearly half of the sample reported at least some sexual activity at some point during active treatment. While a higher proportion of breast cancer patients in our study reported experiences of desire and arousal at 12 months post chemotherapy initiation than gynecologic cancer patients, there were no significant differences in sexual satisfaction ratings between the two groups. When comparing patients based on sexual activity, those who reported at least some sexual activity were more likely to report experiencing sexual desire and arousal and also reported higher sexual satisfaction than sexually inactive participants 12 months after initiating chemotherapy.

Sexual activity during treatment predicted the presence of desire and arousal as well as higher sexual satisfaction 12 months post chemotherapy initiation. Thus, it appears that remaining sexually active during treatment may be associated with better sexual function and higher sexual satisfaction in the year following initiation of chemotherapy. However, it is important to note that this was not a randomized study design and that the relationships observed may be bidirectional in nature. Given that our models controlled for the presence of sexual desire and arousal at the start of chemotherapy, these findings point to the relationship between being sexually active during treatment and potentially higher sexual functioning. While baseline sexual satisfaction was also associated with satisfaction ratings 12 months after chemotherapy initiation, sexual activity remained a significant predictor when these baseline ratings were included in the exploratory models.

The majority of patients who were sexually inactive during treatment did not report resuming sexual activity within 1 year following chemotherapy treatment initiation, pointing to the possible difficulties female cancer survivors have in resuming sexual activity after prolonged cessation. Further, being sexually inactive for the entire year following chemotherapy initiation was associated with higher baseline rates of depressive symptoms and lower relationship satisfaction ratings, pointing to the likely bidirectional relationship between psychosocial variables and sexual functioning.

Sexual activity may serve as a protective factor against treatment-related side effects, such as loss of vulvovaginal lubrication and elasticity [33] by maintaining blood flow and moisturization of the vaginal tissue. Refraining from sexual activity during chemotherapy may increase the likelihood that future attempts are more painful and unpleasant, creating a difficult feedback loop [2, 34]. While results of this study suggest that patients might benefit from engaging in sexual activity during treatment in an effort to disrupt this cycle, it is yet unclear whether providers should provide recommendations for maintaining sexual activity during chemotherapy treatment as part of their standard treatment education. However, this study suggests that it may not be beneficial for providers to outright discourage such activity. Further, any education or recommendations regarding sexual activity during chemotherapy would also need to account for individual patients’ preferences and well-being around sexuality as well as alternatives to sexual activity such as use of dilators and moisturizers to address symptoms.

While it is useful to know that engagement in sexual activity predicts better outcomes on average, being sexually active does not guarantee better sexual function or higher satisfaction. Engagement in sexual activity may lead participants to be more acutely aware of treatment-related changes in sexual function, such as problems with lubrication and orgasm. The relationship between sexual activity and sexual satisfaction is complex and highly personal, with non-sexual factors often holding high subjective importance [35–37]. Given that sexual inactivity during the entire study period was associated with higher baseline depression symptoms, pre-existing emotional distress may be important factors to consider when determining risk for long-term sexual inactivity following cancer treatment.

Previous research has shown that some patients fear that engaging in sexual activity during treatment could be detrimental to their or their partner’s health [23]. It is therefore important that healthcare providers raise the topic of sexual health with their patients and address such misconceptions. When it comes to making specific recommendations for coping with the sexual health side effects of chemotherapy, this study suggests that recommendations might center around tools to help patients remain sexually active (e.g., recommending moisturizers and/or lubricants for vaginal dryness), rather than encouraging patients to take a break from sex. Such an approach would be more consistent with recommendations for other chemotherapy toxicities such as fatigue [21, 38] and peripheral neuropathy [20, 39] and could be incorporated into more comprehensive conversations about activity recommendations for cancer patients during active treatment and later into survivorship. Further, it will be important for psychosocial providers to address sexual concerns among cancer patients, both during active treatment and throughout survivorship. Psychosocial care may benefit from addressing concerns about sexual activity specifically during chemotherapy through pre-existing models (e.g., PLISSIT or EX-PLISST) [40] or the development of novel psychosocial interventions. Specifically, the findings of this study point to the importance of patient education about the safety of sexual activity during chemotherapy, cognitive restructuring of patient’s thoughts and fears about sexual activity during chemotherapy to help with sexual concerns, as well as the potential impact on depression and sexual satisfaction.

This study was limited in that it was conducted as a secondary analysis from a larger study focused on coping and broader chemotherapy side effects, not just sexual function. As such, the present study had limited questions about sexual activity and functioning. The sexuality assessment in the larger study was brief, using single items without more specific questions about different types of sexual activity, and may limit the generalizability of these findings to the wide variety of sexual activities people may engage in, both with and without a partner. Further, the dichotomous categorization of participants as either sexually active or inactive limits our ability to investigate how differing levels of sexual activity may be related to functioning. However, doing so enabled us to combine the questions about partnered and unpartnered sexual activity to better represent both types of sexual activity. Assessment of sexual function via dichotomous items also limits the interpretation of findings, but it is nonetheless an important starting point for understanding the benefits of sexual activity during treatment and likely more representative of how these concerns are queried in oncology practices. Future studies would benefit from assessing sexual outcomes, including sexual activity, functioning, and satisfaction, using more comprehensive instruments to offer a more complete and nuanced view of the complex relationship between sexual activity and function during cancer treatments. In addition, data on sexual histories and outcomes before primary care treatment (e.g., surgery) were unavailable. Collecting these earlier data may provide a better understanding of the additional impact of cancer diagnoses, symptoms, and surgery on sexual function. In the larger study, the research team instructed participants to complete the baseline sexuality items based on experiences prior to symptom onset; however, this may have been difficult for participants depending on the possible chronicity and ambiguity of some symptoms, especially among gynecologic cancer patients. In addition, this study used a small sample size that was mostly White and heterosexual and could not account for all possible covariates, which may limit the reliability or generalizability of the findings to larger and more diverse patient populations with a wide variety of experiences; however, the exploratory findings are an important first step in better understanding the relationship between sexual activity during chemotherapy and sexual outcomes a year after chemotherapy initiation. Future studies would benefit from larger and more diverse samples to have more robust effect sizes that better reflect diverse patient populations. We also acknowledge that missing data is a limitation in this study for both the main and exploratory analyses. Further, the larger study investigated participant functioning across the natural course of chemotherapy treatment and did not assess engagement in supportive treatments/services for physical and/or emotional concerns. Thus, we were unable to control for such treatment participation in this secondary data analysis. Finally, this study did not assess factors that may be related to sexual functioning and satisfaction, such as trauma history or religious/cultural considerations. Future studies may benefit from including these to provide a more complete view of participant’s sexual functioning.

## Conclusion

Overall, these results suggest that engaging in sexual activity during chemotherapy may be associated with better sexual outcomes among female breast and gynecologic cancer survivors, particularly in the domains of desire and arousal. Descriptions of this sample reveal that breast and gynecologic cancer survivors who are sexually inactive during chemotherapy are likely to remain inactive up to 12 months after chemotherapy treatment initiation. Future research should investigate additional predictors of sexual function and satisfaction after cancer treatment in female survivors to develop treatment recommendations that address sexual concerns.

## Data Availability

Data is provided upon request to the coresponding author.
